# Interdisciplinarity at the nexus of biomedical science training: The R3 Center for Innovation in Science Education

**DOI:** 10.1016/j.isci.2025.112735

**Published:** 2025-06-05

**Authors:** Ilinca I. Ciubotariu, Tamaki Kobayashi, Crystal J. Neely, Daniel Fiifi Tawia Hagan, David Ewusi-Mensah, David Van Vactor, Elizabeth C. Whipple, Emma Camacho, Gautam Ghosh, Heather M. Lamb, Isaac Owusu-Frimpong, James Alltop, Julia D. Romano, Kristen Kelly, Lindsay Smith Rogers, Lymari Morales, Matthew Seibel, Roshni Rao, Soumia Bekka, William T. Mills, Arturo Casadevall, Gundula Bosch

**Affiliations:** 1 Department of Molecular Microbiology and Immunology, Bloomberg School of Public Health, Johns Hopkins University, R^3^ Center for Innovation in Science Education, Baltimore, MD, USA; 2 Doctoral and Postdoctoral Life Design, Johns Hopkins University, Baltimore, MD, USA; 3 Department of Environment, Ghent University, Ghent, Belgium; 4 Eco Amet Solutions Limited, Accra, Ghana; 5 Green Living Chats Podcast, Accra, Ghana; 6 Blavatnik Institute of Cell Biology, Harvard Medical School, Boston, MA, USA; 7 Biological and Biomedical Sciences Graduate Program, Harvard Medical School, Boston, MA, USA; 8 Informationist Services, Welch Medical Library, Johns Hopkins University, Baltimore, MD, USA; 9 Ohio State Biochemistry Program, The Ohio State University, Columbus, OH, USA; 10 Department of Molecular Microbiology and Immunology, Bloomberg School of Public Health, Johns Hopkins University, Baltimore, MD, USA; 11 Communications and Marketing, Office of External Affairs, Bloomberg School of Public Health, Johns Hopkins University, Baltimore, MD, USA; 12 Department of Science, Mount St. Mary’s University, Emmitsburg, MD, USA

## Abstract

The R^3^ Center for Innovation in Science Education (R^3^ISE), established at Johns Hopkins Bloomberg School of Public Health, addresses critical gaps in scientific education by instilling the core values of rigorous research, reproducible methods, and scientific responsibility in our students. Through graduate- and professional-level courses, certificate programs, workshops, and open-access resources, R^3^ISE fosters critical thinking, communication, leadership, and other skills essential for scientists.

In this Backstory piece, faculty, students, alumni, and network partners reflect on their experiences with R^3^ISE, which were highlighted in the past year’s annual symposium. In this symposium, themes such as ethical leadership, translating classroom theory into practice, and strategies to combat misinformation highlighted R^3^ISE sustained efforts and proposed further directions. These reflections exemplify how the R^3^ISE community continues to grow—fostering scientific integrity, resilience, and empathy within a global network committed to excellence and responsibility in the biomedical sciences.

**Figure:**
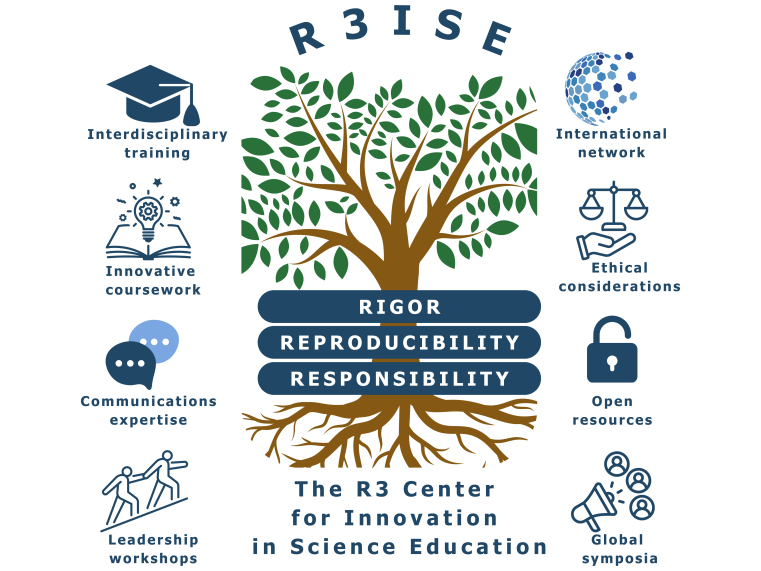
Above image: General graphical representation of the R3 Center for Innovation in Science Education's core values and impact.

The R^3^ISE Center focuses its initiatives on the development of graduate-level training programs and coursework, workshops, and symposia aimed at embedding these R^3^ core norms of open science and good research practice across all levels of scientific inquiry.

We argue that this form of graduate science education would produce more broadly trained scientists capable of conducting deep and rigorous research and connecting the dots between disciplines.

The Katharine E. Welsh symposium provides a platform for scientist-educators and students to come together and promote thought exchange across disciplines.

The symposium showcased the Johns Hopkins R^3^ISE Center’s commitment to fostering an environment where ethical considerations stand at the forefront of scientific inquiry and education.

Since 2017, we have built a global network of R^3^ educators and partner institutions to join us in our efforts to improve science education, and with that, scientific practice.

## Main text

The R^3^ Center for Innovation in Science Education (R^3^ISE) (https://publichealth.jhu.edu/the-r3-center-for-innovation-in-science-education) at the Bloomberg School of Public Health (BSPH), Johns Hopkins University, stands as a beacon for interdisciplinarity in graduate STEM education with its emphasis on the three norms (R^3^) of scientific research: *rigor of research conduct*, *reproducibility of scientific methods and findings*, *and responsibility of scientists to society*.^1^ Founded by Dr. Arturo Casadevall and established by Dr. Gundula Bosch in 2017, the R^3^ISE Center focuses its initiatives on the development of graduate-level training programs and coursework, workshops, and symposia aimed at embedding these R^3^ core norms of open science and good research practice across all levels of scientific inquiry.^2^ Following several groups’ and agencies’ assessments of problems in the scientific enterprise, recommendations to improve training, and calls for educational reform at the graduate level, numerous institutions across the US and globally have embarked on a journey to adopt our approach or adapt it, and have made efforts to change instruction and enhance scientific rigor.^3^^,^^4^^,^^5^^,^^6^^,^^7^^,^^8^^,^^9^ Among our international R^3^ network partners, the R^3^ program at BSPH was among the first to be established and fully implemented, with a strong focus on science’s fundamental principles, interdisciplinary curricula and dialogue across disciplines and career stages.^10^^,^^11^^,^^12^

Our approach for reforming graduate education was also rooted in the observation that oftentimes, established graduate programs fall short in imparting essential skills training in key competencies—critical, interdisciplinary, and creative thinking—that are vital for preparing graduates to navigate the complexities of the modern scientific workforce.^1^^,^^9^^,^^10^ Hence, our curriculum is designed to allow students to acquire a background in immunology and infectious diseases research, and concurrently develop skills in critical thinking, philosophy, responsible written and oral prowess, research design, quantitative reasoning, logic, ethics, and critical analyses—all of which are essential to becoming effective communicators and agents of change in any scientific career.^2^^,^^13^^,^^14^

Furthermore, we argue that this form of graduate science education would produce more broadly trained scientists capable of conducting deep and rigorous research and connecting the dots between disciplines, while also limiting some of the issues with biomedical sciences, such as poor reproducibility, shoddy literature, and retracted publications.^1^^,^^2^^,^^10^ The R^3^ program was created to fill this gap and address the fundamental aspects of scientific reasoning and methods to enhance the overall quality and integrity of research. The R^3^ISE Center not only emphasizes research excellence but also ensures that the future scientific workforce is well-prepared to tackle the ethical challenges posed by an ever-evolving research landscape.

Since our program launch, we have expanded it to serve a global community of scientists, now offering a novel, accredited track in rigorous, reproducible, and responsible research investigation in immunology and microbiology (R^3^IM) for PhD trainees, certificate programs for graduate students, workshops on ethics for postgraduates, training sessions for continuing-science educators, and open educational resources for everyone (https://github.com/JHU-R3ISE/R3easoning)—all housed under the BSPH roof. We have also expanded our network and impact globally, with many partner institutions across the US and the world implementing and incorporating core curriculum elements of R^3^ into their respective graduate educational programs.

To further encourage exchange among the R^3^ community and facilitate international discourse, the R^3^ISE center hosts an international Katharine E. Welsh symposium annually (https://publichealth.jhu.edu/the-r3-center-for-innovation-in-science-education/events/katharine-e-welsh-symposium). This event is supported by the late Katharine Welsh, who was one of four pioneering women who studied Immunology and Zoology in the late 1920s, at the W. Harry Feinstone Department of Molecular Microbiology and Immunology, and who later dedicated her career to the Baltimore City Health Department Laboratories. Each year, the Katharine E. Welsh symposium provides a platform for scientist-educators and students to come together and promote thought exchange across disciplines. Furthermore, each iteration of the symposium has a unique theme centered around the values of the R^3^ISE Center as graduate science education faces a range of challenges and opportunities in R^3^ training. Hence, the symposia cover topics relevant to continuing the work toward improving graduate science education, including bridging the gap between classroom and research practice; integration of up-to-date, professional skill-training in elements like logical fallacies; translation of communication and leadership in training; debunking misinformation and fighting disinformation. In brief, the format includes presentations, interactive discussions, and panels, through which experienced scientist-educators provide examples and strategies for tackling issues related to R^3^ in their institutional training programs. Furthermore, students from R^3^-network institutions share their takeaways from R^3^ training and showcase their integration of principles into application, ranging from the laboratory setting to medical practice and beyond.

### 2024 symposium

#### Day 1: Translating R^3^ education into practice and application

This past October, the symposium with the motto, *The 3R’s Across the Curriculum in Health, Research, and Science Education*, unfolded over two half-days, each packed with insights into how R^3^ principles guide facets of various biomedical careers and academic culture. The theme of the first day centered around the R^3^ principles in training across disciplines and practices. Thus, the emphasis was placed on trainees, highlighting present and past students of R^3^ and-related training, and how they have translated these values into research, professional, and career settings. The day began with an exploration of biomedical careers and how R^3^ integrates into personal and professional development. The keynote presentation covered aligning career trajectories with core competencies and ethical frameworks, stressing the importance of an “internal compass” in navigating the biomedical field. A diverse panel comprising students and alumni from various institutions then delved into the application of R^3^ across different disciplines, illustrating the broad relevance of these principles. Presentations ranged in topic from integrating the 3Rs into laboratory work, to developing undergraduate teaching material, to the role of R^3^ principles in shaping medical practice.

#### Day 2: Integrating R^3^ into communication and leadership for impact

The second day of the symposium revolved around the motifs of scientific communication and leadership across various forms of outreach and impact. The day kicked off with a powerful narrative on leadership and advocacy in science, emphasizing the role of self-advocacy in effective leadership. The subsequent session addressed the nuances of scientific communication, with contributions from professionals with experiences across avenues of communication ranging from journalism to content strategy, podcasts and blogs, in public health and other disciplines. One important topic of discussion included strategies to combat misinformation and enhance communication in scientific settings. A townhall discussion provided a dynamic platform for covering the interplay between leadership, civility, and outreach in scientific communities, stressing the importance of clear and respectful communication. Throughout the course of the symposium, it became apparent that the values of R^3^ could be expanded to include resilience and empathy.

#### Symposium reflections: Expanding the horizon of R^3^

(1) The symposium not only reinforced the importance of the R^3^ in academic and professional realms but also showcased the Johns Hopkins R^3^ISE Center’s commitment to fostering an environment where ethical considerations stand at the forefront of scientific inquiry and education.
(2) Attendees, ranging from current and past students to seasoned professionals, engaged in thoughtful dialogue on enhancing the integrity and impact of research through these guiding principles.
(3) Through panels, keynote speeches, and interactive discussions, the symposium served as a critical reminder of the ongoing need to embed the R^3^ values deeply into the fabric of scientific research and education—ensuring that they resonate across all levels of academic and professional pursuits in the biomedical sciences.
(4) We emphasize that our network is ever-expanding, and we wanted to highlight the contributions of our global partners and students by sharing some direct input below from selected key players.

#### Reflections

##### What are your experiences implementing an R^3^-like program at your institution?

*David Van Vactor*: Bringing together scientists and educators across the Harvard Medical School community to discuss innovations and standards of practice for training in research rigor, reproducibility, and responsibility, sparked a lively exchange and much cross-fertilization of ideas for the future. The effort helped us appreciate how much excellent work and expertise we have around us, both locally in our school and affiliates, and globally in so many of our peer institutions. The effort tapped into our scholarly instincts and has fostered many new initiatives.

##### In what ways did the interdisciplinary nature of the R^3^-focused courses you completed shape your perspective on your research?

*Gautam Ghosh*: The R^3^-focused courses have deeply shaped my research outlook as data grows exponentially in large-scale experiments. They reinforced the need for rigorous methodologies to ensure data quality, responsibility in handling and interpreting vast datasets in an ethical manner, and reproducibility to validate findings across studies. This foundation is essential for maintaining integrity and reliability in the era of large-scale data-driven research.

*Isaac Owusu-Frimpong*: The R^3^ course was a great eye-opener for me, especially since my educational background from my home country did not offer organized courses that focus on scientific education—the concept of rigor, reproducibility, and responsibility in scientific research with highlights on the role of serendipity in scientific discovery. As a young scientist conducting research on malaria, it is my *responsibility* to contribute my quota to the fight against the global malaria burden. To achieve this, I undertake *rigorous* experiments on how the *Plasmodium berghei SERA 5* gene and mosquito enolase gene contribute to malaria infection, which provides knowledge on malaria control strategies. Most importantly, I am shaped by the fact that the outcome of the rigorous experiments must be *reproducible* for validation by the scientific community.

*James Alltop*: I think one of the most important ways my R^3^ coursework changed how I approach research was that it reinforced being present and self-aware and not treating our tools as “black boxes”. Research is not an occupation where you can allow things to become routine or yourself to become complacent; when issues arise in things we take for granted, or if we don’t understand how they work, we are poorly equipped to solve them.

*Kristen Kelly*: The R^3^ courses broadened my research perspective by emphasizing the integration of scientific rigor and ethical responsibility. We are taught not only robust experimental design and unbiased data interpretation across many scientific fields, but also the importance of upholding scientific integrity. This has been especially valuable in my work with children living with perinatal HIV, where both scientific accuracy and ethical considerations for the participants whom we are working with are essential for advancing the field in a meaningful and responsible way.

*Matthew Seibel*: The interdisciplinary nature of this program helped me understand the importance of critical thinking and understanding of literature. The importance of rigor and reproducibility allowed me to produce more thorough and rigorous methods for research into antimicrobial research that I developed for my thesis and challenged the current methods in the field. Had I not taken any classes in the R^3^ curriculum, I feel that this understanding would’ve been lost, and I would have had less appreciation for the importance and impact of my work in medical science and public health.

*Soumia Bekka*: The interdisciplinary nature of the R^3^ courses broadened my perspective by encouraging me to view my research through multiple scientific lenses. Learning how different disciplines establish causal relationships and address scientific errors enhanced my ability to critically evaluate my own data and design more robust experiments for studying perinatal HIV-1. Overall, the R^3^ courses strengthened the methodological soundness of my research while also reinforcing the importance of scientific communication and social responsibility in advancing public health research.

##### How have you applied skills or insights relevant to the R^3^ program into your professional (academic, research, clinical, etc.) practice? Which skills were most relevant? How did you use them?

*William T Mills IV*: In my research, I used what I learned about scientific practice and reproducibility to improve a paper I published about a computational program I developed by ensuring that the code was well-annotated, allowed for reproducible results across users, and was freely available to the public. In my teaching, I used what I learned about scientific writing and reading scientific literature to improve the feedback I was able to provide students about their writing and to develop lessons for undergraduates about reading scientific papers and being able to interpret figures.

##### Given your expertise with various forms of leadership and outreach, how have you integrated ethical values such as compassion, civility, and empathy, across your line of work?

*Emma Camacho*: In my position, I have the privilege of playing both the roles of mentor and mentee, which allows me to continually integrate ethical values into my work approach. I take what life throws at me —whether work, family, or surroundings —with a strong interest in whether it is helping me develop and become a better person. I encourage those I mentor to ask themselves: *What can I do to change this situation? Why shouldn’t this event happen to me? What am I supposed to learn from this experience*? In my view, we are free to choose the course of our lives, as beautifully expressed by the poem *The Road Not Taken* by Robert Frost. Through our vocation, every day we have the opportunity to infuse our work with compassion and empathy for the benefit of others, creating meaningful and lasting impact on those around us, and therefore, also on ourselves.

*Heather M. Lamb*: Whether mentoring students in the lab or connecting with colleagues to build community, I find that good listening is key for developing meaningful connections with others and for understanding their values and motivations. Such awareness allows me to adapt my leadership style to best support the needs of my students and colleagues with the goal of creating an inclusive, supportive environment in which everyone thrives. This also builds a foundation of trust that is critical for fostering community and connection.

*Julia D. Romano*: In the course “Civility, Inclusion, and Professionalism” we stress ethical values when dealing with the complications of the workplace. Workplace conflict can arise from differences of opinion, which are healthy and can lead to growth, but often arise from communication issues. Either way, to have a productive discussion it is important to not only proceed with civility, but to have empathy and compassion toward the other party to be able to effectively understand their point of view.

*Roshni Rao*: Ethical leadership requires addressing challenges with empathy and creating space for growth. When a team member struggled to meet deadlines, I worked with them to uncover the underlying issues, build their confidence, and realign priorities. This not only resolved the immediate challenge but also reinforced trust, showing that leadership is as much about development as it is about outcomes.

##### How can we responsibly utilize our roles as leaders to effectively communicate across audiences?

*Daniel Fiifi Tawia Hagan* and *David Ewusi-Mensah*: History has shown that the most effective leaders—those who successfully guide groups toward a common goal—are those who inspire their audiences to truly care about their message, regardless of differing backgrounds or perspectives. This connection is achieved when leaders genuinely strive to understand their audience’s cultural contexts, skill sets, and struggles, which form the foundation of meaningful dialogue. So, just as all our communication devices operate on a listen-and-speak pattern, effective communication also relies on feedback; leaders must actively listen to their audience’s responses, including nonverbal cues, to gauge understanding and engagement. Furthermore, leaders should remain flexible, adapting their communication style if the audience appears disengaged or unpersuaded, recognizing that the same message may resonate differently with various groups.

*Lindsay Smith Rogers*: A major responsibility is to answer questions that people might have. Otherwise, we’re just talking at our audiences, which is not effective. We also have a duty to help inform people of important context and nuance. We’re not just trying to get the right information out there, it’s crucial to think about improving an audience’s literacy and critical thinking skills to help them seek out trustworthy sources.

*Lymari Morales*: Leaders play an important role in setting the tone for an organization internally and shaping its brand externally. How we communicate is critical in both respects. Communication should be approachable, accessible, and empathetic. Put more simply: we should speak as real humans to real humans in terms they understand and mindful of their varied experiences and perspectives. Listening is also very important to informing our communications.

##### How do you think elements of today’s frequently observed mis- and dis-information across platforms impact critical thinking tendencies in science?

*Daniel Fiifi Tawia Hagan* and *David Ewusi-Mensah*: Misinformation and disinformation on social media, coupled with the rise of short-form content, have diminished people’s ability to engage deeply with complex scientific ideas. While concise communication is beneficial, it often leads to a loss of interest in philosophical and critical thinking, as well as overconfidence in superficial knowledge. This trend undermines the careful evaluation of arguments and thoughtful questioning essential for meaningful scientific inquiry, posing challenges for the future of critical thinking and problem-solving in science.

*Lindsay Smith Rogers*: The same elements that can fuel viral content—attention grabbing, salesmanship, and emotion provoking—can also erode or even counterbalance critical thinking. When competing for eyes and ears, content creators may utilize these tactics without thinking through the fact that they might be fearmongering, misleading, or even totally incorrect. As likes, clicks, and shares pile up, algorithms favor this incendiary content which may drown out factual content that, quite frankly, usually isn’t as catchy.

*Lymari Morales*: Misinformation and disinformation definitely complicate the communications environment and pose a lot of risk. It’s important to share credible, valuable information and get it to the platforms where misinformation and disinformation can prosper. It’s also important to correct misinformation and to refute disinformation when we can.

##### In which ways are classical norms of R^3^, such as good stewardship and transparency, important for the objectives of open science?

*Elizabeth C. Whipple*: Informationists partner with faculty on research projects; we are interested in the reproducibility of searches and making sure those searches are available as underlying data for published papers. Additionally, within our profession, we also will solicit peer review of searches from other information professionals to make sure to not miss something important. This is one example of how informationists have a role in stewardship of information, reproducibility, and transparency.

*Ilinca I. Ciubotariu*: The classical norms of R^3^—rigor, responsibility, and reproducibility—are essential to open science because they ensure research integrity, foster trust, and promote collaboration. Good stewardship encourages careful data management and sharing, making research accessible and reusable, while transparency enhances reproducibility by enabling others to verify and build upon findings. In the context of the R^3^ISE program at Johns Hopkins, which emphasizes these principles in scientific research and graduate education, these norms support a culture of openness and accountability. By embedding stewardship and transparency into research practices, R^3^ISE helps advance open science, fostering innovation and trust across the broader scientific community.

##### What are the benefits of integrating R^3^ values into professional development training?

*Crystal J. Neely*: Responsibility to society is a crucial aspect of professional development, ensuring scientists not only conduct rigorous and reproducible research but also effectively communicate their findings and its impact on health and society. With so much misinformation circulating, this goes beyond speaking with fellow researchers. It’s essential that scientists have the ability to discuss their work with family, policymakers, and strangers alike.

*Tamaki Kobayashi*: Rigor, reproducibility, and responsibility are often assumed to be inherent in our education and training, yet we must remain mindful of them. Embracing the 3Rs involves self-reflection and critical thinking, which are essential for personal and professional growth. By fully embracing the 3Rs, we empower ourselves to navigate challenges and transform them into avenues for innovation and leadership. The notion of transforming challenges into opportunities resonates deeply with me, as it embodies the essence of growth, adaptability, and progress.

##### What strategies can be implemented to further integrate the R^3^ principles into broader learning environments (i.e., undergraduate level, medical field, etc.)?

*Daniel Fiifi Tawia Hagan* and *David Ewusi-Mensah*: We believe that for the R^3^ principles to truly become an integral part of a person’s character, they must be^1^ introduced at an early stage and^2^ taught in a way that emphasizes their significance. Introducing these principles early could involve integrating them into high school curricula, or even earlier, by encouraging teachers to incorporate them into learning activities. Equally important is fostering understanding by consistently explaining the importance of the R^3^ principles at each life stage while seeking feedback from children on how they perceive their value. This approach nurtures a genuine appreciation for the principles, enabling individuals to practice them effortlessly and thoughtfully, even in the face of challenges.

*William T Mills IV*: To further integrate R^3^ principles into broader learning environments, educators can build R^3^ lessons into their current coursework to show students how these principles apply to their specific disciplines. Additionally, R^3^ practitioners can develop virtual, open-access, on-demand content that prospective students can make use of at any time, rather than having to enroll in formal paid courses to be exposed to the material and learn these principles.

##### What was your inspiration behind the R^3^ program?

*Arturo Casadevall*: “Current PhD programs are excellent at teaching students how to do deep work and that needs to be protected and encouraged. However, current programs are not very good in teaching critical thinking or developing broadly trained scientists. The goal of the R^3^ program is to maintain the rigorous training in laboratory research while also teaching didactically the fundamentals of good science, rigor, communication, etc … The R^3^ program is currently focused on PhD training but we hope that some of the principles that we are trying to develop, such as teaching critical thinking, could be applicable to other disciplines such as medicine.”^15^

##### Looking forward, what do you envision for the expansion of R^3^ or similar programs and what improvements should be the focus next?

*Gundula Bosch*: We are observing an organic growth of the R^3^ program initiative on several levels. We began with programs and certificates on the graduate level (Doctoral and Master’s), for the most part in health, biomedicine, and a range of other STEMM disciplines. Since 2017, we have built a global network of R^3^ educators and partner institutions to join us in our efforts to improve science education, and with that, scientific practice.

Currently, we are expanding into the professional and post-graduate arena by offering train-the-trainer leadership and mentoring courses, as well as advanced, continuing scientific education workshops and events for professional researchers from all sectors in the scientific enterprise. Participants gain opportunities to discuss R^3^-related successes and challenges of their own research experiences and are formally trained to teach “R^3^” at their home organization. Together with partner institutions, we prepare to expand into undergraduate-level and high school education in the near future.

#### Resource sharing

The R^3^ISE Center is committed to the principles of open science and the sharing of resources, embodying a forward-thinking approach to research transparency and accessibility. We support and encourage the dissemination of scientific knowledge and educational materials to the wider community. We have previously shared resources from our program in earlier publications and platforms like GitHub (https://github.com/JHU-R3ISE). We have also created a YouTube channel (https://www.youtube.com/@R3ISE-u9h), where the videos from this past symposium and future events such as workshops, etc. will be made publicly available.

## Acknowledgments

We would like to extend our thanks to all of the members of our R^3^ISE network for their continued support and contributions to our growing community. We’d also like to acknowledge the sources of support in the form of grants, etc. for our program. We specifically thank the Katharine E. Welsh Family Foundation for the support and in particular, for sponsoring the symposium.

## Author contributions

The following authors contributed to the writing and editing of the original manuscript: I.I.C., G.B., and T.K. Contributions in the form of quotes were provided by all authors. The manuscript was reviewed and approved by all authors.
